# Comprehensive Clinicopathologic and Molecular Analysis of Mast Cell Leukemia With Associated Hematologic Neoplasm: A Report and In-Depth Study of 5 Cases

**DOI:** 10.3389/fonc.2021.730503

**Published:** 2021-09-13

**Authors:** Philippa Li, Giulia Biancon, Timil Patel, Zenggang Pan, Shalin Kothari, Stephanie Halene, Thomas Prebet, Mina L. Xu

**Affiliations:** ^1^Department of Pathology, Yale School of Medicine, New Haven, CT, United States; ^2^Section of Hematology, Department of Internal Medicine and Yale Comprehensive Cancer Center, Yale School of Medicine, New Haven, CT, United States

**Keywords:** mast cell leukemia, associated hematologic neoplasm, whole-exome sequencing, acute myeloid leukemia, systemic mastocytosis (SM)

## Abstract

Mast cell leukemia with associated hematologic neoplasm (MCL-AHN) is a rare and highly aggressive entity that remains understudied due to the paucity of cases. We present a case of a 45-year-old man who was concurrently diagnosed with mast cell leukemia and acute myeloid leukemia. We identified four additional patients who had MCL-AHN in our institution and performed whole-exome sequencing of all available tumors. Our series revealed a novel and identical *NR2F6* variant shared among two of the patients. This case series and sequencing results demonstrate the importance of fully characterizing rare tumors that are resistant to treatment.

## Introduction

Mast cell leukemia (MCL) is an exceedingly rare and aggressive form of systemic mastocytosis (SM), carrying a poor prognosis with median survival time of less than 6 months (1, 2). The diagnostic criteria of MCL require mast cell (MC) involvement of >20% of the bone marrow aspirate and can be further classified into typical/classic MCL (≥10% MCs in peripheral blood) or aleukemic variant (<10% MCs in peripheral blood) (1). MCL can arise *de novo* or by transformation from other SM subtypes (1). Furthermore, it can be diagnosed with an associated hematologic neoplasm, so-called MCL-AHN.

Somatic gain-of-function mutations in the coding region of *KIT* are identified in >80% of SM, most frequently *KIT* D816V (1). Unfortunately, despite this frequently identified targetable mutation, SM is often refractory to medical treatment. Given the advancements in molecular studies, more complex genetic profiles have been described in SM. We present comprehensive clinicopathologic features of five cases of MCL-AHN coupled with results from in-depth targeted DNA sequencing and whole-exome sequencing (WES). In addition to mutations in *KIT* and other genes associated with hematologic malignancies, our series revealed a novel and identical *NR2F6* variant shared among two of the patients. This advocates for the integration of high-throughput genomic profiling to better understand the molecular landscape of this disease process.

## Case Description

Patient 1 is a 45-year-old man with no significant past medical history. He presented to the hospital with abdominal pain and malaise of 2 weeks. Laboratory studies were notable for macrocytic anemia (10.6 g/dl, reference range 12.0–18.0 g/dl; 102.0 fl, reference range 78.0–94.0 fl) and profound thrombocytopenia (19,000/μl, reference range 140,000–440,000/μl). Initial bloodwork also identified 33% blasts in the peripheral blood. Physical exam was notable for a palpable spleen to the left umbilicus with mild tenderness to palpation. An ultrasound examination demonstrated an enlarged spleen measuring 22.2 × 21.0 cm without focal lesions or perisplenic collections. The patient was subsequently admitted to the hematology service for further workup.

**Figure 1:**
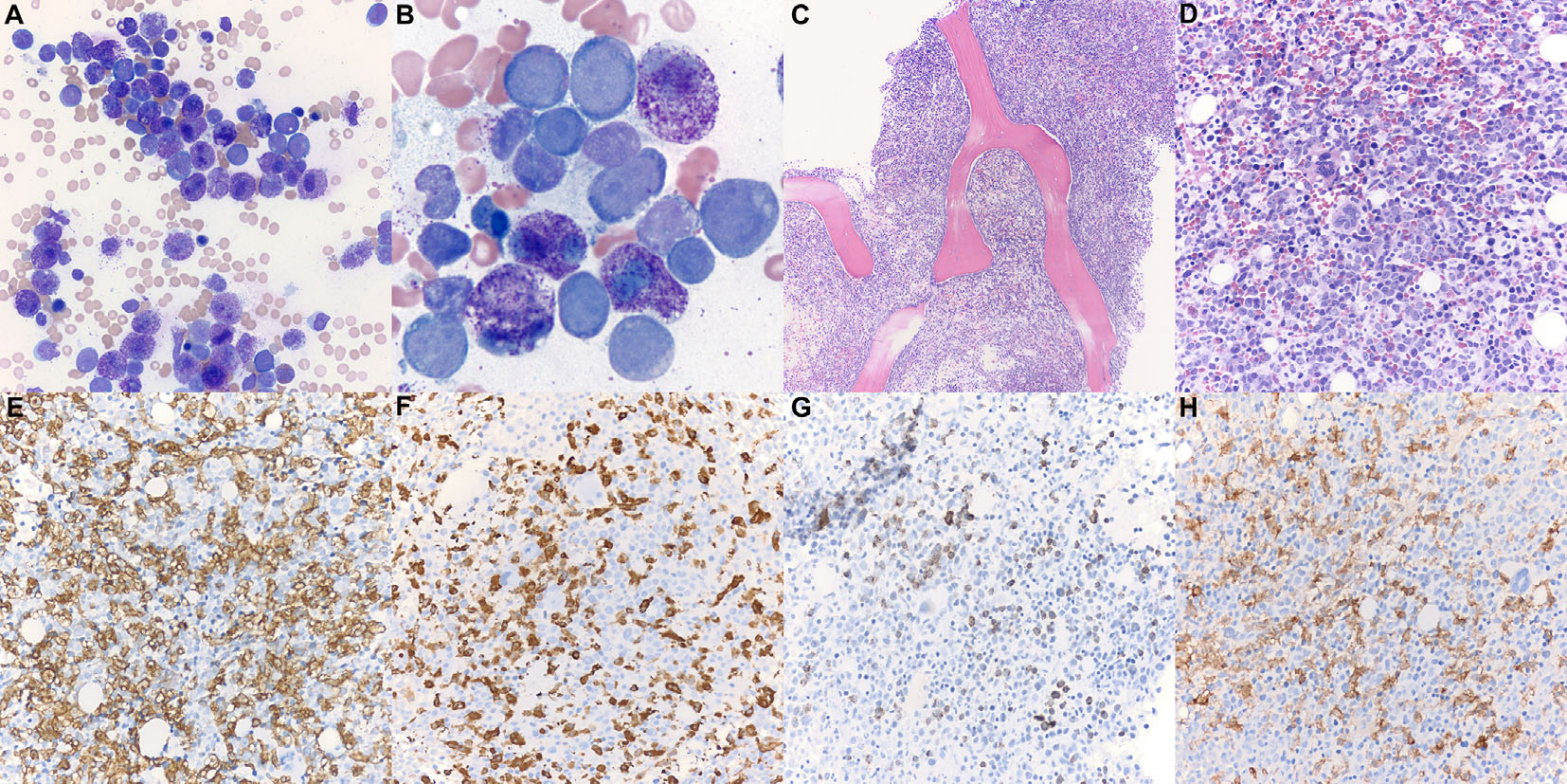
Patient 1 bone marrow aspirate and trephine biopsy. **(A, B)** Bone marrow aspirate smear (Wright-Giemsa, ×400, ×1,000) is notable for numerous mast cells intermixed with myeloblasts. Degranulated mast cells are interspersed throughout the bone marrow aspirate smear. **(C)** The bone marrow biopsy (H&E, ×100) demonstrates hypercellular marrow for age (90% cellularity). **(D)** Approximately 50% of the cellularity is composed of mast cells with oval nuclei, and hypogranulated cytoplasm (H&E, ×400). Blast elements and other marrow elements are scattered throughout. **(E)** CD117 immunostain (×400) highlights abundant mast cells. **(F)** Mast cell tryptase immunostain (×400). **(G)** CD34 immunostain (×400) highlights blast elements, comprising >20% of non-mast cell cellularity. **(H)** CD25 immunostain (×400) is positive in the neoplastic mast cells.

Bone marrow biopsy (**Figure 1**) demonstrated markedly hypercellular marrow (90% cellular), with approximately 50% of the cellularity composed of pale, round, degranulated mast cells scattered and in clusters, aberrantly expressing CD25. Aspirate smears revealed approximately 48% abnormal degranulated mast cells and 23% myeloblasts. The patient was diagnosed with mast cell leukemia, aleukemic variant, with concurrent acute myeloid leukemia (MCL-AML). His serum tryptase level was 614 μg/l (reference range <11.0 μg/l) at the time of diagnosis.

The patient received chemotherapy with 7 + 3 regimen (cytarabine 100 mg/m^2^/day and idarubicin 12 mg/m^2^) with midostaurin (50 mg daily). After salvage cladribine (0.13 mg/kg daily for 5 days) with high-dose midostaurin, his recovery marrow showed persistent mast cell leukemia with no increase in blasts. The patient was subsequently switched to avapritinib, and his tryptase levels decreased from 391 to 22 UI/l after 6 months. The patient’s bone marrow at 8 months following initial diagnosis showed 15% mast cells without evidence of AML, compared to 50% mast cells at initiation of avapritinib. He received a matched unrelated donor peripheral blood stem cell transplant 10 months after initial diagnosis, which was complicated by severe gastrointestinal graft-versus-host-disease. Currently, the patient is 17 months from his initial diagnosis; he remains on avapritinib with regular follow-up.

Four additional patients with MCL-AHN were identified within our institution, and all available records were reviewed (**Table 1**). Patient 2 was a 71-year-old man who presented for evaluation of extreme fatigue. Workup revealed early T-cell precursor acute lymphoblastic leukemia (ETP-ALL), and the patient was started on combination chemotherapy consisting of 6-mercaptopurine, vincristine, methotrexate, and prednisone (POMP) with low-intensity intrathecal methotrexate. A posttreatment bone marrow biopsy/aspirate 3 months later demonstrated no evidence of residual leukemia but marked mastocytosis (63%) with aberrant positive expression of CD25 and CD2, consistent with MCL. Flow cytometry revealed recurrent ETP-ALL 19 months after the first diagnosis of malignancy despite maintenance chemotherapy. He died of disease 2 months later.

**Table 1:** Summary of MCL-AHN cases.

	Patient 1	Patient 2	Patient 3	Patient 4	Patient 5
Age (at diagnosis of first hematologic malignancy), race, and gender	45 yo Caucasian male	71 yo Caucasian male	74 yo Caucasian male	60 yo Caucasian male	68 yo Caucasian female
MCL type	Aleukemic *de novo* MCL	Aleukemic *de novo* MCL, subsequent diagnosis	Aleukemic secondary MCL, subsequent diagnosis	Aleukemic secondary MCL, subsequent diagnosis	Aleukemic *de novo* MCL, subsequent diagnosis
AHN type	AML without maturation	ETP-ALL	MDS with ringed sideroblasts	AML NOS	AML with t(8;21)
Aspirate findings at diagnosis	23% blasts and 48% mast cells	31% lymphoblasts on initial aspirate, 63% mast cells on aspirate	3% myeloblasts on initial aspirate, 20% mast cells on subsequent aspirate	78% myeloblasts and <5% mast cells on initial aspirate, 37% mast cells on subsequent aspirate	72% myeloblasts on initial aspirate, 34% mast cells on subsequent aspirate
Cytogenetics	Trisomy 8q, trisomy 21	Normal 46, XY male karyotype	Normal 46, XY male karyotype	Failed cytogenetics	Positive *RUNX1T1*/*RUNX1* fusion
S/A/R status^‡^	S/A/R^pos^	Unknown	S/A/R^neg^	Unknown	S/A/R^neg^
Outcome	Alive	Deceased	Deceased	Deceased	Deceased
Interval time between MCL and AHN	Concurrent diagnoses	3 months	9 years 10 months	10 months	2 months
Survival time from first diagnosed malignancy	17 months	21 months	14 years 3 months	21 months	47 months

MCL, mast cell leukemia; AHN, associated hematologic neoplasm; AML, acute myeloid leukemia; ETP-ALL, early T-cell precursor acute lymphoblastic leukemia; MDS, myelodysplastic syndrome; ^‡^ S/A/R status indicates any mutation present in panel of SRSF2, ASXL1, and RUNX1.

Patient 3 was a 74-year-old man who was evaluated 14 years prior for chronic anemia. His bone marrow biopsy at that time revealed myelodysplastic syndrome with ringed sideroblasts, and he was managed on observation. Eight years later, a repeat marrow biopsy was performed for worsening anemia, demonstrating persistent myelodysplastic syndrome and 3% mast cells. He was started on Aranesep and red blood cell transfusions. Repeat bone marrow 2 years later revealed persistent myeloid neoplasm with mast cell aspirate count of 20%, consistent with MCL-AHN. He began midostaurin and received darbepoetin every other week. The patient died 4 years after the diagnosis of MCL-AHN from pneumonia with persistent mastocytosis at time of death.

Patient 4 was a 60-year-old man who presented to the hospital with a suspected cardiovascular accident. His initial bone marrow biopsy demonstrated AML with abnormal mast cells (positive for CD25 and CD2) involving <5% marrow cellularity. He was treated with standard induction chemotherapy followed by consolidation. A repeat marrow biopsy 10 months later demonstrated no evidence of AML but 37% mast cells consistent with MCL. He received cladribine, then switched to midostaurin for persistent disease. The patient failed to respond significantly to any MCL therapy, and subsequent bone marrow biopsies continued to show persistent MCL without evidence of AML. He died of disease less than 2 years after his initial diagnosis.

Patient 5 was a 68-year-old female who was diagnosed with AML after presenting with fatigue and increased peripheral blood myeloblasts. The patient was started on standard induction chemotherapy and achieved morphologic remission on day 14. One month later, a bone marrow aspirate revealed 11% myeloblasts with 34% mast cells, which aberrantly expressed CD25. The patient underwent consolidation therapy with a matched unrelated donor stem cell transplant. Unfortunately, the patient demonstrated AML relapse and persistent low-level mastocytosis. She died of disease 47 months after her initial diagnosis of AML.

## Methodology

Available specimens from all five MCL-AHN patients were subjected to molecular profiling: allele-specific PCR for *KIT* mutation on residue 816, targeted DNA-sequencing for genes relevant in myeloid neoplasms, and/or whole-exome sequencing (WES) combined with a high-coverage spike-in panel for known cancer-associated genes (see **Supplementary Methodology**). Patient 1 specimens were pretreatment bone marrow (BM), with involvement by MCL-AML, and buccal swab as matched normal sample. Patient 2 specimen was peripheral blood (PB) with involvement by ETP-ALL 1 month prior to the diagnosis of MCL, and PB at MCL diagnosis. Patient 3 and 4 specimens were PB at the time of MCL diagnosis. Patient 5 pretreatment PB was drawn at diagnosis of AML, while her subsequent BM sampled 3 months later was remission AML, with involvement by MCL only (**Tables 1**, **2**).

**Table 2:** Molecular profiling results.

**Patient 1**		
Timepoint	MCL-AHN	
Source	BM	
Molecular Method	targeted DNA-seq, WES	
Variants:		
***KIT*** c.2447A>T p.D816V, COSMIC: COSV55386424, dbSNP: rs121913507	32.6	
***PHF6*** c.1024C>T p.R342X, COSMIC: COSV59699091, dbSNP: rs132630297	13.7	
***RUNX1*** c.319C>T p.R107C, COSMIC: COSV55866866	45.6	
**Patient 2**		
Timepoint	ETP-ALL	MCL-AHN
Source	PB	PB
Molecular Method	WES	PCR
Variants:		
***KIT*** c.2447A>T p.D816V, COSMIC: COSV55386424, dbSNP: rs121913507	n/d	present
***DHRS4L2*** c.236A>G p.E79G, COSMIC: COSV58730199	2.4	** **
***DNMT3A*** c.2656C>G p.Q886E, COSMIC: COSV53044466, dbSNP: rs752280049	15.9	
***EXOC7*** c.1826G>A p.R609Q, COSMIC: COSV58035445, dbSNP: rs768692750	50.0	
***JAK2*** c.1849G>T p.V617F, COSMIC: COSV67569051, dbSNP: rs77375493	4.3	
***NR2F6*** c.394C>G p.P132A, COSMIC: COSV52244108, dbSNP: rs202200760	47.8	
***PTPN11*** c.179G>T p.G60V, COSMIC: COSV61005028, dbSNP: rs397507509	5.9	
**Patient 3**		
Timepoint	MCL-AHN	
Source	PB	
Molecular Method	targeted DNA-seq	
Variants:		
***KIT*** c.2447A>T p.D816V, COSMIC: COSV55386424, dbSNP: rs121913507	7.0	
***CBL*** c.1228-2A>G splice acceptor variant, COSMIC: COSV50629953, dbSNP: rs727504426	10.0	
***SF3B1*** c.2098A>G p.K700E, COSMIC: COSV59205318, dbSNP: rs559063155	36.0	
**Patient 4**		
Timepoint	MCL-AHN	
Source	PB	
Molecular Method	PCR	
Variants:		
***KIT*** c.2447A>T p.D816V, COSMIC: COSV55386424, dbSNP: rs121913507	present	
**Patient 5**		
Timepoint	AML	MCL-AHN
Source	PB	BM
Molecular Method	targeted DNA-seq, WES	targeted DNA-seq, WES
Variants:		
***KIT*** c.2447A>T p.D816V, COSMIC: COSV55386424, dbSNP: rs121913507	33.8	n/d
***ANO4*** c.2747G>A p.R916Q, COSMIC: COSV54611712, dbSNP: rs267603265	50.6	39.4
***CACNA1C*** c.5737G>A p.E1913K, COSMIC: COSV59697224, dbSNP: rs200231105	7.3	10.2
***DNM2*** c.1072G>A p.G358R, COSMIC: COSV58965871, dbSNP: rs267606772	4.1	n/d
***GNB1*** c.169A>G p.K57E, COSMIC: COSV66100005, dbSNP: rs141326438	n/d	13.6
***JAK2*** c.1849G>T p.V617F, COSMIC: COSV67569051, dbSNP: rs77375493	4.2	n/d
***NR2F6*** c.394C>G p.P132A, COSMIC: COSV52244108, dbSNP: rs202200760	43.4	35.3
***WFIKKN2*** c.244G>A p.V82I, COSMIC: COSV60958856, dbSNP: rs562819910	54.0	47.6

Variants are defined by: gene, coding sequece change, amino acid chage, COSMIC ID, dbSNP ID if available, variant allele frequency (color-coded according to the values; lowest value in blue, highest value in orange).

For samples with targeted DNA-seq and WES profiling, only the WES frequency is reported.

After KIT variant, the other variants are reported in alphabetical order. Variants identified in more than one sample are underlined.

MCL-AHN, mast cell leukemia with associated hematologic neoplasm; AML, acute myeloid leukemia; BM, bone marrow; WES, whole exome sequencing; ETP-ALL, early T-cell precursor acute lymphoblastic leukemia; PB, peripheral blood; PCR, polymerase chain reaction.

### Targeted Sequencing Panel

Tumor specimens from patients 1 (BM), 3 (PB), and 5 (PB, BM) underwent high-throughput genotyping analysis by the Tumor Profiling Laboratory at Yale New Haven Hospital. Genomic DNA was extracted from the specimens and amplified using 1,298 primer pairs, designed with Ion Torrent AmpliSeq software, covering full exonic regions or hotspot regions of selected genes (see **Supplementary Methodology**). Next-generation sequencing was performed on the Ion Torrent S5 Sequencer.

### Whole-Exome Sequencing

Additional tumor specimens from patients 1 (BM), 2 (PB), and 5 (PB, BM) underwent WES with a high-coverage spike-in panel for known cancer-associated genes developed at the Yale Center for Genome Analysis in collaboration with Integrated DNA technologies (IDT) and the Yale Cancer Center. Tumor DNA was extracted from bone marrow cells or peripheral blood cells after Ficoll separation. Normal DNA for Patient 1 was extracted from buccal swab. DNA extraction was performed using the QIAGEN DNeasy Blood & Tissue Kit according to the manufacturer’s instructions. WES libraries were prepared adding spike-ins for cancer genes and sequenced on the Illumina NovaSeq sequencer (paired-end, 100bp).

## Discussion

To date, the most significant prognostic indicator for MCL patients is mutational status in any of the *SRSF2*, *ASXL1*, or *RUNX1* genes, collectively referred to as S/A/R gene panel (3). In a study cohort of 25 MCL patients, approximately half demonstrated S/A/R^pos^ status (1), which correlated with more aggressive phenotypes, more (intrinsic) resistance to disparate treatment modalities, and worse treatment response in comparison to S/A/R^neg^ patients (1). In our cohort, only patient 1 was S/A/R^pos^ due to a *RUNX1* mutation (**Table 1**). The S/A/R status of patients 2 and 4 is unknown due to limited molecular study results.

The PB sample of Patient 5 was taken at the diagnosis of AML without known evidence of MC disease and contained the following variants: *KIT* p.D816V, *ANO4* p.R916Q, *CACNA1C* p.E1913K, *DNM2* p.G358R*, JAK2* p.V617F, *WFIKKN2* p.V82I, and *NR2F6* p.P132A. Her BM specimen following treatment showed no evidence of leukemia but did contain MCL. This subsequent specimen demonstrated the following variants: *GNB1* p.K57E, *ANO4* p.R916Q, *CACNA1C* p.E1913K, *WFIKKN2* p.V82I, and *NR2F6* p.P132A. Peripheral blood sequencing identified the *KIT* p.D816V mutation, which may have hinted at early/evolving MCL disease. Interestingly, the *KIT* mutation is not identified on the later BM specimen, possibly indicating treatment effect on *KIT*+ mast cells (**Table 2** and **Supplementary Figure 1**).

We also identified identical missense *NR2F6* variants in patients 2 and 5, previously undescribed in SM. *NR2F6* (nuclear receptor subfamily 2, group F, member 6) is an orphan member of the nuclear receptor superfamily (4). This variant is clinically interesting due to studies demonstrating that *NR2F6-/-* mice had enhanced IL-2 and IFN-gamma secretion, favoring T cell-mediated cancer cell elimination (4). *NR2F6*-deficient tumor-bearing mice have enhanced survival *via* T cell-dependent antitumor immunity (4). This is a possible means to potentiate established PD-L1 and CTLA-4 blockade therapies (4, 5). Further studies will be necessary to determine the function of the *NR2F6* p.P132A missense variant observed in these cases, exploring the somatic pathogenicity or the germline predisposition for MCL.

In MCL, *KIT* p.D816V is found in approximately 55% of cases, which is lower in frequency than in other forms of advanced SM (2). Even though all our patients demonstrated the *KIT* p.D816V mutation in at least one specimen (**Table 2**), none of them demonstrated durable response to targeted therapy. The average survival time was 32 months following the diagnosis of MCL. The only patient to have considerable survival time after the first hematologic diagnosis was patient 3, who had a less aggressive AHN with a long interval time prior to the development of MCL.

Avapritinib, a new tyrosine-kinase inhibitor targeting *KIT* and its mutants, is the most recent addition to our drug armamentarium and has shown some remarkable activity in less aggressive presentations (6). Its use in advanced forms of mast cell disease such as MCL is scarce, but the treatment response presented in patient 1 is encouraging.

The direction of hematologic malignancies has increasingly leaned toward molecular profiling, which would aid in the diagnosis, prognosis, and identification of potential novel treatment options. We conducted a pilot study on five patients affected by MCL-AHN. Despite the small size of the patient cohort due to the rarity of the disease and the limited specimen availability given the nature of retrospective studies, the application of a boosted whole-exome sequencing allowed us to detect somatic variants at the whole-exome level without losing depth in regions frequently affected by cancer-associated mutations, resulting in a comprehensive and deep characterization of MCL-AHN molecular features. MCL has proven to be largely resistant to treatment, despite frequently identified *KIT* mutations. The molecular aberrations of MCL-AHN identified presently are not well understood but could be of potential value in treatment guidance.

## Data Availability Statement

The original contributions presented in the study are included in the article/**Supplementary Material**. Further inquiries can be directed to the corresponding author.

## Ethics Statement

The studies involving human participants were reviewed and approved by Yale University IRB. The patients/participants provided their written informed consent to participate in this study.

## Author Contributions

PL, GB, and MX designed and performed the research as well as wrote the manuscript. PL and MX collected patient samples. GB analyzed next-generation sequencing data. TPa, ZP, SK, SH, and TPr revised the clinical data and edited the manuscript. All authors contributed to the article and approved the submitted version.

## Funding

This work was in part supported by the DeLuca Center for Innovation in Hematology Research at Yale Cancer Center and The Frederick A. Deluca Foundation.

## Conflict of Interest

MX serves as a consultant (advisory board) for Blueprint Medicines and for Seattle Genetics.

The remaining authors declare that the research was conducted in the absence of any commercial or financial relationships that could be construed as a potential conflict of interest.

## Publisher’s Note

All claims expressed in this article are solely those of the authors and do not necessarily represent those of their affiliated organizations, or those of the publisher, the editors and the reviewers. Any product that may be evaluated in this article, or claim that may be made by its manufacturer, is not guaranteed or endorsed by the publisher.

## Abbreviations

MCL, mast cell leukemia; SM, systemic mastocytosis; MC, mast cell; MCL-AHN, mast cell leukemia with associated hematologic neoplasm; AML, acute myeloid leukemia; ETP-ALL, early T-cell precursor acute lymphoblastic leukemia; WES, whole-exome sequencing; BM, bone marrow; PB, peripheral blood.
