# Supplementary material for: Comprehensive Clinicopathologic and Molecular Analysis of Mast Cell Leukemia With Associated Hematologic Neoplasm: A Report and In-Depth Study of 5 Cases
Source: Front Oncol. 2021 Sep 13;11:730503. doi: 10.3389/fonc.2021.730503 (PMC8474637; doi:10.3389/fonc.2021.730503)
Supplement: Supplementary file 1 [file DataSheet_1.docx]

**Supplementary Methodology**

*Allele-specific Polymerase Chain Reaction (AS-PCR)*

DNA from patients 2 (peripheral blood, PB) and 4 (PB) was isolated and subjected to allele-specific PCR amplification at ARUP Laboratories (www.aruplab.com). The reaction uses an oligonucleotide primer set specific for the exon 17 of *KIT* on chromosome 4, and an allele-specific primer that specifically initiates amplification from the allele containing the point mutation in codon 816. Each assay includes a positive control reaction using DNA from a plasmid that contains the *KIT* D816V mutation and a negative control using placental DNA. PCR products are analyzed by electrophoresis and UV transillumination of ethidium bromide stained gels. The D816V mutation can be detected if tumor represents at least 0.3% of cells.

*Targeted sequencing panel - Variant calling and annotation*

Targeted DNA-sequencing data were analyzed (e.g. read alignment, mapping using human reference sequence hg19 and variant calling) with the Torrent Suite Software. Minimum quality scores of Q15 and coverage of 100X were used. Annotation of variants was performed using Ion Reporter Software. Association of variants with previously documented cancer specimens was determined using COSMIC (cancer.sanger.ac.uk/) and ClinVar (www.ncbi.nlm.nih.gov/clinvar/) databases. DNA sequences used as references for this panel of genes can be found at http://www.ncbi.nlm.nih.gov/refseq/rsg/. The mutation nomenclature is based on the convention recommended by the Human Genome Variation Society (http://www.hgvs.org/mutnomen/).

Genes tested (49 genes relevant to myeloid neoplasms):

*ABL1  ALK  ASXL1  ATRX  BCOR  BCORL1  BRAF*  BRCC3  CALR*  CBL  CEBPA  CSF3R  DNMT3A  EED  EP300ETV6  EZH2  FLT3*  GATA1  GATA2  IDH1*  IDH2*  JAK2  KIT  KRAS  MPL  MYC  NF1  NPM1*  NRAS*

*PDGFRA  PDS5B  PHF6  PRPF8  PTPN11  RAD21  RUNX1  SETBP1  SF3B1  SMC1A  SMC3  SRSF2  STAG1*

*STAG2  TET2  TP53  U2AF1  WT1  ZRSR2*

*indicates genes where only 1-3 exons containing hotspot regions were sequenced.

*Whole exome sequencing - Variant calling and annotation*

Raw files were analyzed with FastQC (https://www.bioinformatics.babraham.ac.uk/projects/fastqc/, v0.11.5) for quality control. Sequencing reads were then aligned to the human genome (GRCh38.p10) with Burrows-Wheeler Aligner (BWA-MEM , v0.7.17) (1) obtaining an average of 167,618,578 total aligned reads. Duplicate reads (average of 20.9%) were removed with Picard v2.9.0 (http://broadinstitute.github.io/picard/). Coverage analysis was performed using BEDTools v2.27.1 (2) obtaining an average depth of 275.8X calculated on the median coverage per target region in each tumor sample (Patient 1, tumor sample: 240.0X, normal sample: 178.0X; Patient 2, tumor sample: 317.0X; Patient 5, tumor sample at diagnosis: 303.0X, tumor sample after treatment: 243.0X).

Single-nucleotide variants (SNVs) and indels were detected with GATK4 Mutect2 (3). We applied the tumor-normal variant calling workflow for Patient 1, with genomic DNA extracted from buccal swab as matched normal sample, and the tumor-only variant calling workflow for Patients 2 and 5, using a “Panel of Normals” (PoN) available to download as part of the GATK resource bundle, due to the absence of matched normal samples. Variants were functionally annotated using ANNOVAR (2019Oct24) (4).

High-confidence variants were filtered as follows: exclusion of false positives (technical artifacts, sequencing errors and germline variants) through GATK4 filtering using default parameters; exclusion of variants with synonymous or unknown effect; selection of deleterious variants (FATHMM-MKL prediction score) (5); annotation in COSMIC v90 and v91 as confirmed somatic variants (6); occurrence in hematopoietic tissues. All reported variants were visually inspected with the Integrative Genomics Viewer (IGV) (7) and confirmed with FreeBayes v1.2.0 (8) as additional variant caller. Identified variants were also evaluated through the Single Nucleotide Polymorphism Database (dbSNP, https://www.ncbi.nlm.nih.gov/snp/) and through the Genome Aggregation Database (gnomAD, https://gnomad.broadinstitute.org). High-confidence variants presenting total population frequency >1% were included only if reported by both GATK4 and FreeBayes variant callers (*NR2F6* p.P132A variant, gnomAD reported frequency: 3.5%, 1000Genomes reported frequency: 1.9%).

**References:**

1. Li H, Durbin R. Fast and accurate long-read alignment with Burrows–Wheeler transform. Bioinformatics. 2010;26(5):589-95.

2. Quinlan AR, Hall IM. BEDTools: a flexible suite of utilities for comparing genomic features. Bioinformatics. 2010;26(6):841-2.

3. Benjamin D, Sato T, Cibulskis K, Getz G, Stewart C, Lichtenstein L. Calling Somatic SNVs and Indels with Mutect2. bioRxiv. 2019:861054.

4. Wang K, Li M, Hakonarson H. ANNOVAR: functional annotation of genetic variants from high-throughput sequencing data. Nucleic Acids Research. 2010;38(16):e164-e.

5. Shihab HA, Gough J, Cooper DN, Stenson PD, Barker GL, Edwards KJ, et al. Predicting the functional, molecular, and phenotypic consequences of amino acid substitutions using hidden Markov models. Hum Mutat. 2013;34(1):57-65.

6. Tate JG, Bamford S, Jubb HC, Sondka Z, Beare DM, Bindal N, et al. COSMIC: the Catalogue Of Somatic Mutations In Cancer. Nucleic Acids Res. 2019;47(D1):D941-D7.

7. Robinson JT, Thorvaldsdóttir H, Wenger AM, Zehir A, Mesirov JP. Variant Review with the Integrative Genomics Viewer. Cancer Research. 2017;77(21):e31-e4.

8. Garrison E, Marth GT. Haplotype-based variant detection from short-read sequencing. arXiv: Genomics. 2012.
