# Supplementary material for: Comprehensive Clinicopathologic and Molecular Analysis of Mast Cell Leukemia With Associated Hematologic Neoplasm: A Report and In-Depth Study of 5 Cases
Source: Front Oncol. 2021 Sep 13;11:730503. doi: 10.3389/fonc.2021.730503 (PMC8474637; doi:10.3389/fonc.2021.730503)
Supplement: Supplementary file 2 [file DataSheet_2.docx]

**Supplementary Figure 1**


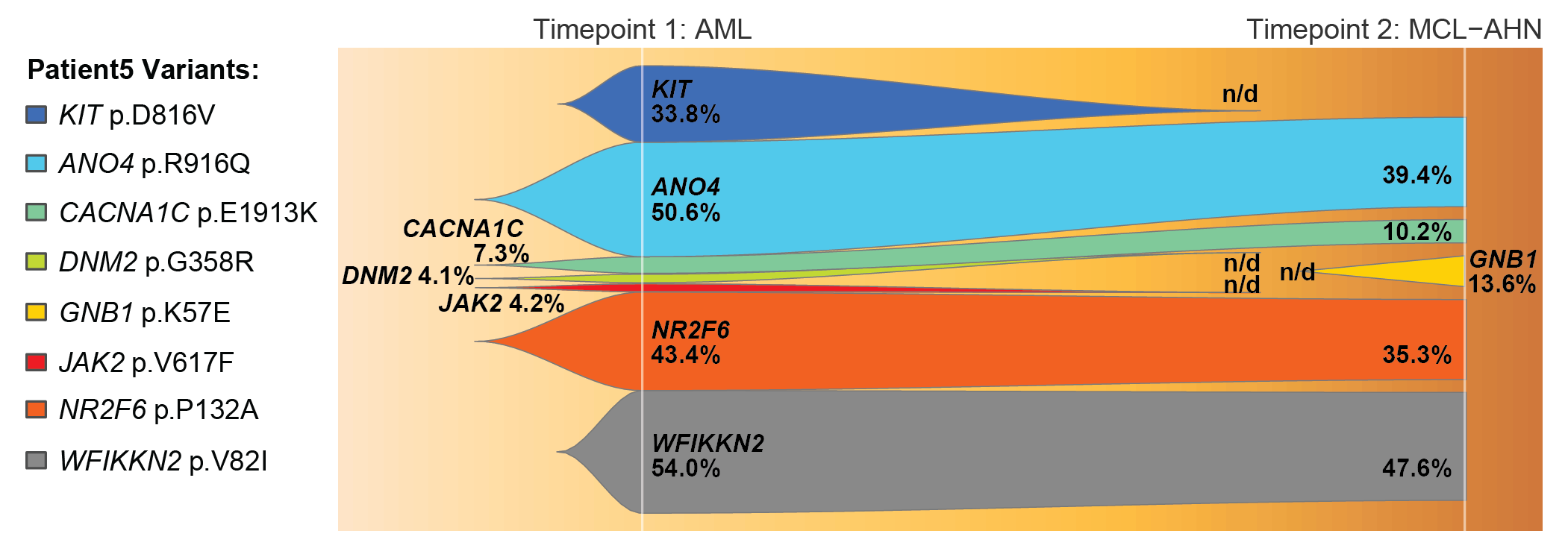


**Schematic of molecular changes in Patient 5 over time.** The plot displays variants exclusively detected in one of the two timepoints (n/d not detected) and changes in the frequencies (%) of variants detected in both timepoints. Timepoint 1 and 2 correspond to the diagnosis of acute myeloid leukemia (AML) and mast cell leukemia with associated hematologic neoplasm (MCL-AHN), respectively. The variant allele frequencies were calculated through whole-exome sequencing analysis. The plot was generated using *fishplot* R package.
